# Preparation and Characterization of Quaternized Chitosan Coated Alginate Microspheres for Blue Dextran Delivery

**DOI:** 10.3390/polym9060210

**Published:** 2017-06-07

**Authors:** Kuo-Yu Chen, Si-Ying Zeng

**Affiliations:** Department of Chemical and Materials Engineering, National Yunlin University of Science and Technology, Yunlin 64002, Taiwan; M10515035@yuntech.edu.tw

**Keywords:** alginate, quaternary ammonium chitosan, graft polymerized, microspheres, drug release

## Abstract

In this study, 2-[(Acryloyloxy)ethyl]trimethylammonium chloride was graft polymerized onto chitosan (CS) to form quaternary ammonium CS (QAC) by using ammonium persulfate as a redox initiator. Alginate (ALG) microspheres loaded with a water-soluble macromolecular model drug, blue dextran (BD), were obtained by corporation of coaxial gas-flow method and ionic gelation process. CS and QAC were then coated on the surfaces of ALG microspheres to generate core/shell structured CS/ALG and QAC/ALG microspheres, respectively. The experiment result showed that QAC/ALG microspheres had a smaller particle size due to the stronger electrostatic interactions between QAC and ALG molecules. In vitro drug release studies at pH 7.4 and pH 9.0 exhibited that the release rate of BD was significantly decreased after ALG microspheres coating with CS and QAC. Moreover, ALG microspheres coated with QAC showed a prolonged release profile for BD at pH 9.0. Therefore, QAC/ALG microspheres may be a promising hydrophilic macromolecular drug carrier for a prolonged and sustained delivery.

## 1. Introduction

Much attention has been given to stimuli-sensitive hydrogels for pharmaceutical and medical applications, such as controlled-release carriers for drug [1,2], protein [3], and vaccine [4] delivery and as scaffolds for tissue engineering [5,6]. Hydrogels could be designed to respond to external stimuli, such as pH and temperature [7]. The pH sensitive hydrogels with ionizable groups have been extensively investigated for drug delivery systems. The drug release rates of polyelectrolyte hydrogels strongly depend on the pH of medium and ionic strength [8]. Alginate (ALG), a natural anionic polysaccharide derived from marine brown algae, has attracted a wide range of biomedical applications because of their low cost, biocompatibility and biodegradability [9]. ALG microspheres prepared by cross-linking with calcium ions to form stable network have been widely used as carriers for the controlled release of drugs and active agents [10,11].

Chitosan (CS), another natural derived polysaccharide, is a biodegradable, non-toxic and biocompatible polymer that has been widely applied in drug delivery systems. In acidic media (pH < 6.5), CS becomes a cationic polymer due to protonation, which could form polyelectrolyte with negatively charged ALG by electrostatic interactions. Coating of ALG microspheres with CS layer could prolong drug release in physiological environment compared with ALG microspheres [3,12] However; changes in the pH of the environment can influence the degree of ionization of CS, resulting in increased or decreased electrostatic interactions between CS and ALG.

Previous study indicated that the quaternized CS increased positive charge density and enhanced solubility in neutral and basic environments [13]. The positive charge characteristic of quaternary ammonium cation is independent of the pH of the aqueous medium [14]. Furthermore, ALG beads coated with quaternized CS containing a higher degree of substitution significantly decreased the release of brilliant blue in 0.9% (*w*/*v*) NaCl [15]. However, a high burst release of around 30% was still discovered in 2 h when quaternized CS had the highest degree of substitution (95%). The molecular structure of CS will affect its binding with ALG. These quaternary ammonium groups of quaternized CS were very close to the CS backbone. Interaction between the quaternary ammonium groups of quaternized CS and carboxylate groups of ALG may be lessened due to steric repulsions between the two molecules. It is interesting to prepare quaternized CS with long pendant side-chains and large charge density by graft copolymerization, which could reduce the intermolecular and intramolecular hydrogen bonds of CS and increase the water solubility of CS. The flexible side-chains with plenty of quaternary ammonium cations on the rigid CS backbone was expected to extend in a plurality of directions and interact with various different ALG molecules through electrostatic interactions, resulting in a highly cross-linked layer. Moreover, the positive charge characteristic of quaternary ammonium cations is independent of the pH of their surrounding medium. The amino (–NH_2_) and hydroxyl (–OH) groups present on the CS backbone can be grafted. The grafting copolymerization of CS with a variety of cationic monomers using redox initiators has been reported in literature [16]. To the best of our knowledge, however, there has been no report on ALG microspheres coated with cationic graft copolymer of CS, and its use as a drug carrier.

Blue dextran (BD) is a stable, high-molecular-weight and water-soluble dextran with covalently bonded blue dye [17]. BD was chosen as a water-soluble biomacromolecular drug model because of its ease of analysis by UV-vis spectrometry [10,18].

In this study, a cationic graft copolymer (QAC) of CS graft-polymerized with quaternary ammonium-containing monomers was synthesized. ALG microspheres were prepared by corporation of coaxial gas-flow technique and ionic gelation process. Then, ALG microspheres were coated with CS and QAC to fabricate CS/ALG and QAC/ALG microspheres, respectively. To investigate the in vitro drug release behavior, BD was used as a high-molecular-weight and hydrophilic model drug to discuss the controlled release of ALG, CS/ALG and QAC/ALG microspheres in different pH medium.

## 2. Materials and Methods

### 2.1. Materials

Sodium alginate was obtained from Acros Organics (Geel, Belgium). CS with molecular weight of 50–190 kDa and deacetylated degree of 75–85% was purchased from Sigma-Aldrich (St. Louis, MO, USA). 2-[(Acryloyloxy)ethyl]trimethylammonium chloride (AETMAC, 80 wt % in water) and blue dextran with molecular weight of 2000 kDa were also obtained from Sigma-Aldrich. Ammonium persulfate was supplied from Showa Chemicals (Tokyo, Japan). Calcium chloride (CaCl_2_), acetic acid, hydrochloric acid (HCl) and sodium hydroxide (NaOH) were obtained from Katayama Chemical (Osaka, Japan). Acetone and methanol were bought from Echo Chemical (Miaoli, Taiwan). Phosphate buffer solution was from UniRegion Bio-Tech (Hsinchu, Taiwan). All reagents were used as received with no additional purification. Aqueous solutions were prepared using deionized water.

### 2.2. Synthesis of Quaternary Ammonium CS (QAC)

QAC was synthesized by the graft polymerization of a cationic monomer, AETMAC, onto CS in aqueous acetic acid with a redox initiator system of ammonium persulfate. Briefly, a 1% (*w*/*v*) acid aqueous solution of CS was prepared by dissolving CS powder in 2% (*v*/*v*) aqueous acetic acid at 80 °C in a four-neck flask equipped with a mechanical stirrer, condenser, nitrogen inlet and addition funnel. The CS solution was purged with nitrogen for 30 min. Then, 0.015 M AETMAC and 0.015 M ammonium sulfate were added successively dropwise to initiate graft polymerization under nitrogen atmosphere. After reaction for 3 h at 80 °C, the reaction mixture was cooled down to room temperature and poured into excess of acetone. The precipitate was filtered off, washed with methanol to remove unreacted AETMAC monomer and poly[2-(acryloyloxy)ethyl]trimethylammonium chloride (PAETMAC) homopolymer. Finally, the resultant product was dried in a drying oven.

### 2.3. Preparation of BD-Loaded ALG, CS/ALG and QAC/ALG Microspheres

ALG loaded with hydrophilic model drug, BD, were obtained by using a coaxial nitrogen flow encapsulator and ionic gelation method [19,20]. CS and QAC were then coated on the surfaces of ALG to generate core/shell structured, respectively. Briefly, a 1.2% (*w*/*v*) aqueous solution of ALG was prepared by dissolving sodium alginate in deionized water. BD was then added into the ALG solution and mixed homogeneously with a magnetic stirrer. The weight ratio of BD to ALG was 1:10. BD-loaded ALG solution was injected through a needle with an inner gauge diameter of 0.17 mm into 5% (*w*/*v*) aqueous CaCl_2_ solution. The particle size of microspheres was controlled by regulating the extrusion flow rate using a syringe pump (KDS-100, KD Scientific, Holliston, MA, USA) at a flow rate of 5 mL/h and by applying a coaxial nitrogen flow using a Var J1 encapsulation unit (Nisco, Zurich, Switzerland) at a rate of 4 L/min. The obtained microspheres were incubated in in the CaCl_2_ solution for 30 min at room temperature to completely cross-link, and were washed gently with deionized water for two times to remove BD attached on the microsphere surface.

CS and QAC were dissolved in 1% (*w*/*v*) aqueous acetic acid solution to prepare 0.5% (*w*/*v*) CS and QAC solutions, respectively. The pH of CS and QAC solutions was adjusted to 6.3 with 1 N NaOH solution. BD-loaded ALG microspheres were then added drop-wise into CS and QAC solutions, stirred for 30 min and washed with deionized water to produce BD-loaded CS/ALG and QAC/ALG microspheres, respectively. The theoretical weight ratio of CS/ALG or QAC/ALG was fixed at 1:1. The actual weight ratio of CS/ALG or QAC/ALG was calculated as: (*W*_d_ − *W*_s_)/*W*_s_, where *W*_d_ is the dry weight of BD-loaded CS/ALG or BD-loaded QAC/ALG microspheres and *W*_s_ is the dry weight of BD-loaded ALG microspheres.

### 2.4. Characterizations

Proton nuclear magnetic resonance (^1^H NMR) spectra were collected on an Agilent Technologies DD2 NMR spectrometer (Santa Clara, CA, USA) operating at 600 MHz. CS and QAC samples were dissolved in 1% (*v*/*v*) CD3COOD in D_2_O before NMR test. The D_2_O peak was used as a reference peak.

Infrared survey spectra of dry CS and QAC within the range 4000–500 cm^–1^ were recorded on a Fourier transform infrared spectrophotometer (FTIR; Bio-Rad FTS-40, Hercules, CA, USA) operated with a dry air purge. The dry sample was mixed with KBr and compressed into thin disks. The spectra were collected at resolution of 2 cm^−1^ and by averaging 32 scans.

The cationic degree (CD) of QAC was determined by the precipitation titration, which depended on the amount of AETMAC units incorporated into the copolymer [21]. Briefly, 0.04 g of dry QAC was dissolved in 50 mL of deionized water, and 1 mL (5% (*w*/*v*)) of aqueous potassium chromate was added as indicator. The solution was titrated with 0.01 M aqueous silver nitrate. The endpoint of titration was reached until the color changed to brick red. The CD of QAC was calculated as follows: CD (%) = {[193.67 × *c* × (V − *V*_0_)]/(1000 × *W*)} × 100%, where *c* (mol/L) is the concentration of aqueous silver nitrate; *V* (mL) and *V*_0_ (mL) are the volumes of the consumed aqueous silver nitrate for titration of QAC and the blank, respectively; *W* (g) is the weight of QAC; and 193.67 (mol/g) is the molecular weight of AETMAC.

Wide-angle X-ray diffraction (WAXD) measurements were performed on CS and QAC using a Rigaku MiniflexII X-ray diffractometer (Tokyo, Japan) equipped with Cu-*K*α radiation over a range (2θ) of 2°–50°, at a scan rate of 2° min^−1^.

Furthermore, the shape of prepared microspheres was examined under an optical microscopy (M835, Microtech; Eugene, OR, USA) equipped with a CCD camera. The average diameter of microspheres was measured from several optical microscopy images and using image analysis software MICROCAM (M&T Optics, Taipei, Taiwan).

### 2.5. In Vitro Drug Release

#### 2.5.1. Drug Encapsulation Efficiency

The drug encapsulation efficiency (EE) of the microspheres was evaluated according to the method reported in previous literature [10]. The actual BD loading amount was calculated indirectly by the difference between the initial amount of BD dissolved in ALG solution and the BD released into the gelling medium and washing solutions. The absorbance of the clear supernatant was measured using a visible spectrophotometer (SP-830 Plus, Metertech, Taipei, Taiwan) at 620 nm. The amount of BD without encapsulation was obtained by comparison with a BD calibration curve. The EE was calculated using the formula, EE (%) = [(*W*_0_ − *W*_a_)/*W*_0_)] × 100%, where *W*_0_ is the initial amount of BD dissolved in ALG solution and *W*_a_ is the amount of BD lost in the gelling medium and washing solutions. The experiments were performed in triplicate and average values were taken.

#### 2.5.2. Cumulative Drug Release

In vitro BD release studies were conducted in three different pH solutions: aqueous HCl solution of pH 3.0 and PBS of pH 7.4 and 9.0. All release experiments were performed using a shaking bath with a shaking speed of 120 rpm at 37 °C to maintain the microspheres in suspension. The freshly prepared microspheres were dispersed in 40 mL of release media. At predetermined time intervals, 4 mL of sample solution was withdrawn and replaced with an equal volume of fresh solution immediately. The sample solution was centrifuged at 8000 rpm for 1 min. The amount of released BD in the supernatant was determined as described above. The cumulative drug release (CDR, %) of each sample was calculated according to the formula CDR (%) = (*W*_t_/*W*_∞_) × 100%, where *W*_t_ is the cumulative amount of BD released from the microspheres at a given time *t* and *W*_∞_ is the amount of BD initially loaded in the microspheres. All drug release experiments were performed in triplicate and average values were taken.

### 2.6. Statistical Analysis

All quantitative data were expressed as mean ± standard deviation. Statistical analysis was conducted using Student’s *t*-test or one-way analysis of variance (ANOVA), followed by post hoc Fisher’s LSD multiple comparison test. Levels of statistical significance were set at *p* < 0.05.

## 3. Results and Discussion

### 3.1. QAC Characteristics

CS is used in multiple biomedical and pharmaceutical applications. However, it is not dissolved in neutral and alkali aqueous solution due to the strong intermolecular and intramolecular hydrogen-bond interactions. The graft polymerization of cationic monomers on CS chains was performed to increase solubility of CS in neutral and alkaline pH solution.

**Figure 1:**
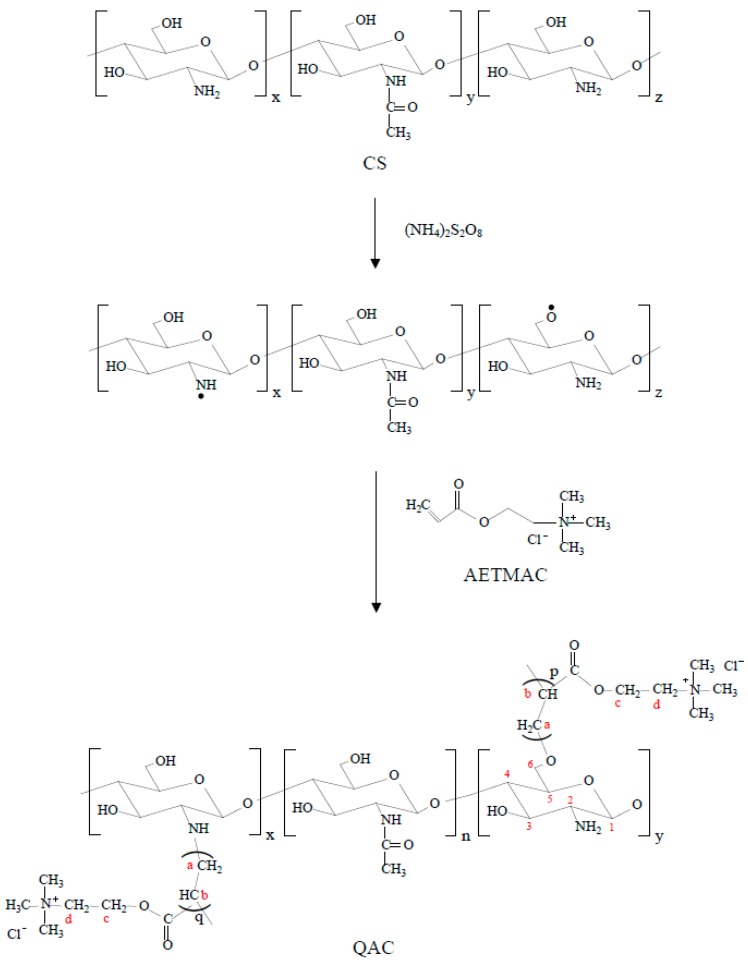
Schematic representation of graft-copolymerization of AETMAC onto CS.

CS has two types of reactive groups, C2–NH_2_ and C6–OH, which can be modified by grafting polymerization method using a redox initiator system [22,23]. The ammonium persulfate could generate the activated radical in the backbone of CS and initiate the reaction [24,25]. Then, the double bond of the AETMAC monomer was polymerized to form the grafted copolymer QAC (Figure 1).

**Figure 2:**
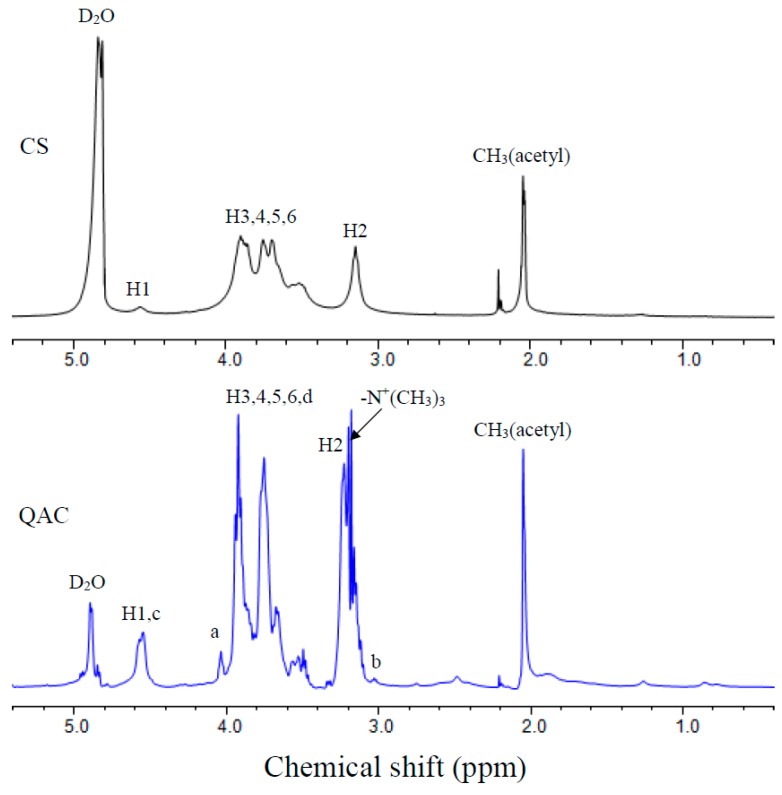
^1^H NMR spectra of CS and QAC.

The chemical compositions and structures of QAC were characterized by NMR, FTIR and WAXD measurements. Figure 2 displays the ^1^H NMR spectra of CS and QAC. The sharp peak appearing at 4.85 ppm is for water. The peaks observed at 4.6, 3.4–4.0, 3.2 and 2.05 ppm correspond to H1, H3,4,5,6, H2 and *N*-acetyl of CS, respectively [21,26]. Compared with CS, the signal observed at 3.18 ppm could be attributed to the nine protons of the quaternary ammonium group, –N^+^(CH_3_)_3_, in PAETMAC [27,28]. Additionally, the signal at 3.0 ppm is ascribed to methine proton (Hb in Figure 1 and Figure 2). The methylene (Ha,c,d in Figure 1 and Figure 2) signals are also observed in QAC. Therefore, the NMR spectrum indicates that CS has grafted with AETMAC.

**Figure 3:**
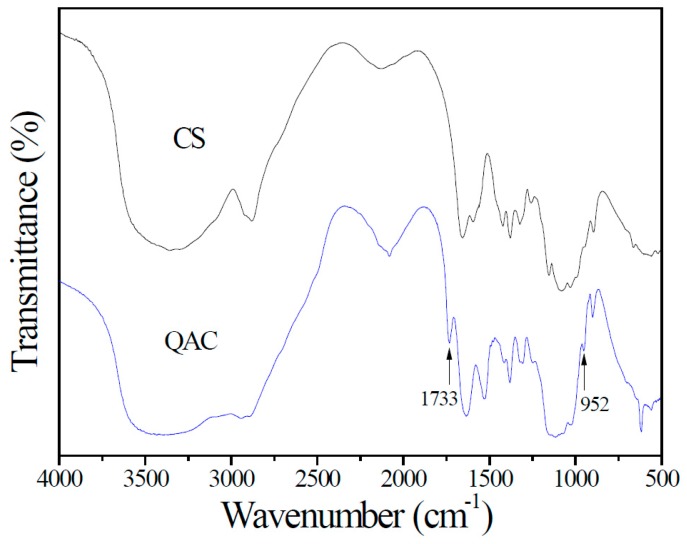
FTIR spectra of CS and QAC.

The FTIR transmittance spectra of CS and QAC are shown in Figure 3. CS and QAC show a characteristic strong and broad band at 3200–3600 cm^−1^ for the O–H and N–H stretching vibrations of amino and hydroxyl groups on the backbone of CS chains. The peak at around 1645 cm^−1^ is ascribed to the C=O stretching vibration of amide I groups. The band at around 1560 cm^−1^ is assigned to the N–H bending and C–N stretching vibration of amide II. In addition to the above-observed bands, QAC shows two additional characteristic absorption bands at 1733 and 952 cm^−1^, which could be resulted from the C=O stretching vibration of ester groups and the C–N stretching vibration of quaternary ammonium groups in PAETMAC, indicating the formation of graft copolymer [16,29]. Moreover, the cationic degree of QAC determined from the precipitation titration analysis is about 21.2% (*w*/*w*), which means that 0.212 g of PAETMAC had been grafted onto per gram of QAC.

**Figure 4:**
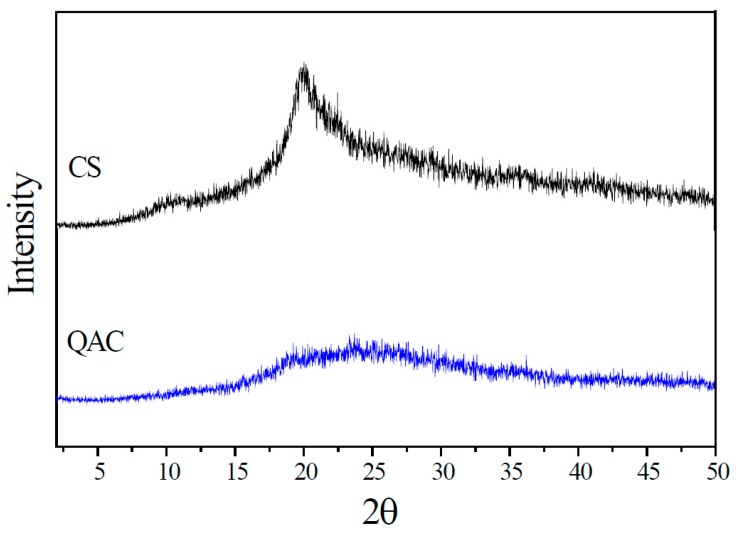
WAXD spectra of CS and QAC.

The crystalline properties of CS and QAC were evaluated by WAXD. As shown in Figure 4, CS has two characteristic diffractive peaks at around 10.3° and 20.2° corresponding to the crystal forms I and II, respectively [30]. However, the intensity of these peaks for QAC sharply decreases and it becomes amorphous. This is attributed to the steric hindrance of the grafted chains: PAETMAC, obstructs the formation of intermolecular and intramolecular hydrogen bonds of the CS backbone, resulting in the disappearance of crystallinity. Therefore, the water solubility of CS would be increased via grafting of PAETMAC on CS chains [31].

On the basis of NMR, FTIR and WAXD analyses, it can be concluded that AETMAC monomers have been successfully grafted onto CS.

### 3.2. Microspheres Characteristics

In this study, the weight ratio of CS to ALG or QAC to ALG was fixed at 1:1 for the preparation of microspheres. However, the actual weight ratios of CS to ALG and QAC to ALG were 0.06:1 and 0.54:1, respectively. Compared to CS, more amount of QAC was coated onto the surface of negatively charged ALG microspheres because QAC had more positive charges than CS at pH = 6.3.

**Figure 5:**
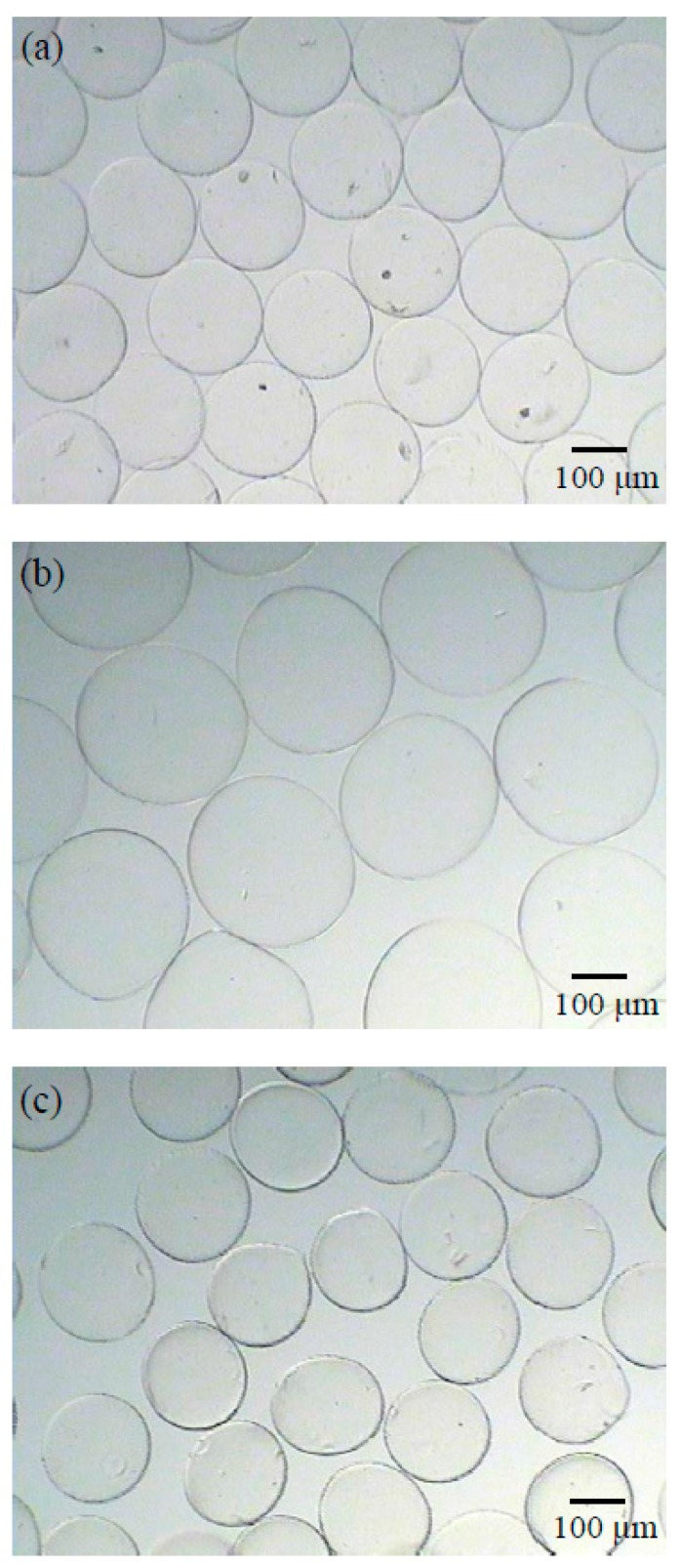
Optical microphotographs of: (**a**) ALG; (**b**) CS/ALG; and (**c**) QAC/ALG microspheres after soaking in deionized water.

Optical microphotographs of wet microspheres and their shape are shown in Figure 5. The ALG microspheres were successfully prepared via the coaxial nitrogen flow technique and ionic gelation method, resulting in a narrow size distribution. As illustrated in Figure 5, all the microspheres had a regular spherical shape and smooth surface. No aggregation was observed. The average diameters of ALG, CS/ALG and QAC/ALG microspheres were 212.9 ± 8.1, 317.5 ± 10.0 and 204.7 ± 7.3 μm, respectively. The coating of CS on ALG microspheres result caused an increase in mean diameter to around 105 μm. However, ALG microspheres coated with QCA led to a decrease in the mean diameter (*p* < 0.05). Although, compared to CS, more amount of QCA was coated onto ALG microspheres, QAC/ALG microsphere had a significantly smaller particle size than CS/ALG microspheres. The difference in the particle size of the microspheres could be due to the difference in the electrostatic interactions occurring between the coating layer and ALG microspheres. QAC has a higher positive charge density than CS at neutral environment, resulting in a stronger electrostatic interaction with ALG. Therefore, QAC/ALG microsphere has the smallest particle size.

### 3.3. BD Release Prolife

ALG microspheres was coated with polycations to decrease the degree of drug burst release from microspheres but minimal effects on drug encapsulation efficiency were desirable. The drug encapsulation efficiency of the ALG, CS/ALG and QAC/ALG microsphere was 54.4% ± 1.5%, 52.4% ± 5.6% and 54.6% ± 3.8%, respectively. There were no significant differences in encapsulation efficiency among the different microspheres (*p* > 0.05). The result indicates that the coating process of CS and QAC on ALG microspheres had no detrimental effect on the drug encapsulation efficiency.

**Figure 6:**
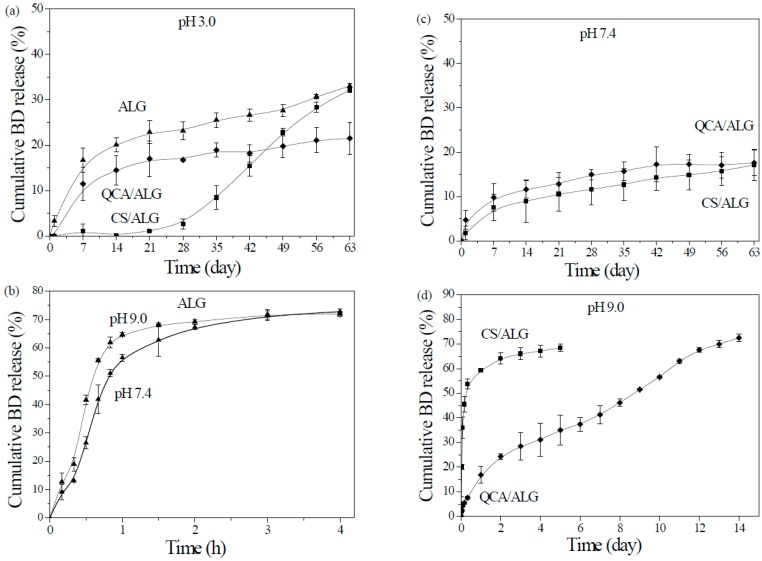
Release profiles of BD from different microspheres at different pH: (**a**) ALG (▲), CS/ALG (■) and QAC/ALG (♦) microspheres at pH 3.0; (**b**) ALG (▲) microspheres at pH 7.4 and 9.0; (**c**) CS/ALG (■) and QAC/ALG (♦) microspheres at pH 7.4; and (**d**) CS/ALG (■) and QAC/ALG (♦) microspheres at pH 9.0.

The BD release behavior from different microspheres was studied in aqueous HCl solution (at pH = 3.0) and PBS (at pH = 7.4 and 9.0). The release profiles of BD from the prepared microspheres are presented in Figure 6. Figure 6a shows the percentage of cumulative BD release at 37 °C from the ALG, CS/ALG and QAC/ALG microspheres in aqueous HCl solution of pH 3.0. About 17% and 11% of total amount of BD were released in seven days from ALG and QAC/ALG microsphere, respectively, and slow release after the initial release was continued. It was clear that the BD release rate of ALG microspheres was much higher than that of QAC/ALG microspheres. The difference in the release rate between ALG and QAC/ALG microspheres become more distinguishable with increasing time. The percentage of cumulative BD release reached 33% for ALG microspheres, whereas the value was only 21% for QAC/ALG microspheres within 63 days. The result suggested that QAC microspheres coating effectively decreased the release of BD in acid environment. In contrast, very low amount (<1%) of BD was released from CS/ALG microspheres during the first 21 days. After 28 days, they started to release rapidly BD up to 32% in 63 days. After 49 days, the amount of BD released from CS/ALG microspheres was higher than that from QAC/ALG microspheres.

Figure 6b shows the release of BD at 37 °C from ALG microspheres in PBS of pH 7.4 and 9.0. A high burst release of around 56% and 65% was discovered for pH 7.4 and 9.0, respectively, in the first hour, followed by a gradual release, reaching about 70% of the loaded amount after 4 h. This result is similar to earlier work. Kim et al. showed that the release of BD from the ALG beads in water was rapid during the first hour, followed by a gradual release up to 3 h [32]. Baimark and Srisuwan prepared ALG microspheres by a water-in-oil emulsion solvent diffusion method [10]. The ALG microspheres were post-cross-linked with calcium ions. They also demonstrated that the release of BD from the ALG microspheres in PBS of pH 7.4 at 37 °C was rapid during the first hour, followed by a gradual release up to 24 h.

It was obvious that the cumulative BD release at pH 7.4 and 9.0 was much higher than that at pH 3.0, suggesting that calcium ion cross-linked ALG microspheres are not stable in neutral and basic environment. ALG microspheres are easily broken in PBS at pH 7.4 because of ion exchange, and therefore their drug release rates are high [33]. ALG is protonated into the insoluble form of alginic acid at pH 3.0, since alginic acid has a p*K*a of 4.25 [34]. Therefore, ALG microspheres had a lower release rate at pH 3.0.

In contrast, ALG microspheres coated with CS and QCA resulted in a small burst release of 5% and 2% in PBS of pH 7.4, respectively, followed by a very slow release reaching 17% of the loaded amount after 63 days (Figure 6c). No significant difference in the degree of release between CS/ALG and QAC/ALG microspheres was founded (*p* < 0.05). The lower burst release and slower release rate indicated that the CS and QAC shells coated on the surface of ALG microspheres could effectively improve their stability at neutral environment, and thus effectively control the release of BD.

CS/ALG microspheres in PBS of pH 9.0 showed a high burst release of around 54% in first 8 h followed by only an additional release of 14% over the next four days (Figure 6d). In contrast, no burst release was observed for QAC/ALG microspheres. The drug release was significantly prolonged to 14 days by the coating of QCA onto ALG microspheres as compared to ALG (4 h) and CS/ALG microspheres (five days). Seventy-three percent of the loaded does was released from QAC/ALG microspheres after 14 days. The p*K*a of CS is approximately 6.5 [35]. Therefore, at pH 9.0, the amino groups of CS were no longer significantly charged due to deprotonation, which weakens the extent of the electrostatic interaction with carboxyl groups of ALG. However, QAC still had permanent positively charged quaternary ammonium groups that interact strongly with ALG. Moreover, more amount of QAC was coated onto the surface of ALG microspheres compared with CS. Therefore, a slower release rate was observed for QAC/ALG microspheres. Although, compared to CS, QAC had no obvious effect in neutral environment, it did significantly affect the release of BD in basic environment.

## 4. Conclusions

In this investigation, quaternary ammonium monomers were graft co-polymerized onto CS using ammonium persulfate as a redox initiator. NMR and FTIR spectra confirmed the formation of graft co-polymer. The ALG, CS/ALG and QAC/ALG microspheres with narrow size distribution and regular spherical shape have been successfully fabrication. The in vitro release study showed that the release of BD from microspheres was pH dependent. Compared to ALG microspheres, QAC/ALG and CS/ALG microspheres could reduce the degree of burst release of BD in neutral and basic environment. Moreover, QAC/ALG microspheres showed prolonged release profiles in basic environment. According to the results of this study, the QAC/ALG microspheres have a potential as a carrier for the pH-controlled release of water-soluble macromolecular drugs.
